# Supplementary material for: Human soluble ACE2 improves the effect of remdesivir in SARS‐CoV‐2 infection
Source: EMBO Mol Med. 2020 Dec 14;13(1):e13426. doi: 10.15252/emmm.202013426 (PMC7799356; doi:10.15252/emmm.202013426)
Supplement: Supplementary file 1 — Appendix [file EMMM-13-e13426-s001.pdf]

Appendix

Human soluble ACE2 improves the effect of remdesivir in SARS-CoV-2 infection

Vanessa Monteil[1], Matheus Dyczynski[2,3], Volker M Lauschke[4], Hyesoo Kwon[5], Gerald Wirnsberger[6], Sonia Youhanna[4], Haibo Zhang[7], Arthur S.Slutsky[7], Carmen Hurtado del Pozo[8,9,10], Moritz Horn[2,3], Nuria Montserrat[8,9,10], Josef M. Penninger[11,12], and Ali Mirazimi[1,5]

Appendix Table S1

Appendix Table S1: p-values for figures 1 and 3

Figure and comparsion	P value
1A non-treated vs 4μM	0.0028
1A non-treated vs 10μM	0.0025
1A non-treated vs 20μM	0.0024
1A non-treated vs 30μM	0.0024
1A 4μM vs 10μM	0.0242
1A 4μM vs 20μM	0.0201
1A 4μM vs 30μM	0.0201
1B non-treated vs hrsACE2 25μg/ml	0.0003
1B non-treated vs hrsACE2 50μg/ml	0.0002
1B non-treated vs hrsACE2 100μg/ml	0.0002
1B non-treated vs hrsACE2 200μg/ml	0.0002
3A non-treated vs hrsACE2	<0.0001
3A non-treated vs Remd. 4μM	<0.0001
3A non-treated vs Remd. 4μM + hrsACE2	<0.0001
3A hrsACE2 vs Remd. 4μM + hrsACE2	0.0364
3A Remd. 4μM vs Remd. 4μM + hrsACE2	0.0023
3B non-treated vs hrsACE2	0.0035

3B non-treated vs Remd. 4μM	0.0035
3B non-treated vs Remd. 4μM + hrsACE2	0.0035
3C hrsACE2 5μg/ml vs hrsACE2 5μg/ml + Remde. 4μM	0.0376
3C Remd. 4μM vs Remd. 4μM + hrsACE2 5μg/ml	0.0055
3C Remd. 4μM vs Remd. 4μM + hrsACE2 10μg/ml	0.0044
3D Non-treated vs Remd. 4μM	0.0003
3D Non-treated vs hrsACE2 10g/ml + Remd. 4μM	0.0003
3D Remd. 4μM vs Remd. 4μM + hrsACE2 10μg/ml	0.0287
3D hrsACE2 10g/ml vs Remd 4μM + hrsACE2 10μg/ml	0.0295
3E non-treated vs hrsACE2 5μg/ml	0.0060
3E non-treated vs hrsACE2 10μg/ml	0.0059
3E non-treated vs hrsACE2 5μg/ml + Remd. 4μM	0.0059
3E non-treated vs hrsACE2 10μg/ml + Remde 4μM	0.0059
3E hrsACE2 5μg/ml vs hrsACE2 10μg/ml	0.0491
3E Remd. 4μM vs Remd. 4μM + hrsACE2 5μg/ml	0.0446
3E Remd. 4μM vs Remd. 4μM + hrsACE2 10μg/ml	0.0443
3F non-treated vs hrsACE2 5μg/ml	0.0021
3F non-treated vs hrsACE2 10μg/ml	0.0020

3F non-treated vs hrsACE2 5μg/ml + Remd. 4μM	0.0019
3F non-treated vs hrsACE2 10μg/ml + Remde 4μM	0.0019
3F Remd. 4μM vs Remd. 4μM + hrsACE2 5μg/ml	0.0235
3F Remd. 4μM vs Remd. 4μM + hrsACE2 10μg/ml	0.0234
